# A Comparison of Two DAAs Used in a Unique Model of Care to Treat Hepatitis C Infections in New Jersey

**DOI:** 10.1093/ofid/ofae645

**Published:** 2024-11-27

**Authors:** Jihad Slim, Paul Bellafiore, Barbara Tempalski, Corey Rosmarin-DeStefano, Kevin Leyden, Juan Torres, Sheena Duprey, Emily Levaggi

**Affiliations:** NYMC, St. Michaels Medical Center, Newark, New Jersey, USA; NYMC, St. Michaels Medical Center, Newark, New Jersey, USA; NJCRI, Newark, New Jersey, USA; NJCRI, Newark, New Jersey, USA; NJCRI, Newark, New Jersey, USA; NJCRI, Newark, New Jersey, USA; NJCRI, Newark, New Jersey, USA; NJCRI, Newark, New Jersey, USA

**Keywords:** glecaprevir/pibrentasvir, hepatitis C, sofosbuvir/velpatasvir

## Abstract

In this prospective observational study, we compare the efficacy of glecaprevir/pibrentasvir vs sofosbuvir/velpatasvir in treating hepatitis C within a unique model of care utilizing a combination of telehealth, an ambulatory van, case management, and a contracted pharmacy. Among 769 patients treated, 90.4% completed treatment, with 9.6% lost to follow-up. Both regimens demonstrated high completion rates and efficacy.

Hepatitis C (HCV) has been a challenging public health concern for many years, and it seems that the issue may be getting worse as both the incidence and prevalence have been increasing in the general population [1, 2]. The prevalence is further increased in difficult-to-reach populations such as those with a history of substance use disorder (SUD). In this population, the estimated prevalence is 39%, representing 6.1 million people [3]. Studies examining why this discrepancy exists have identified some common barriers to initiating care for these individuals such as ongoing drug use, logistical barriers to treatment and medical systems barriers [4]. The World Health Organization (WHO) and the International Network of Hepatitis in Substance Users have developed a framework to help health organizations deliver care to these individuals. The framework focuses on 6 core components: service delivery, health workforce, health information systems, medical procurement, health system financing, and leadership and governance, with a seventh component—communication and engagement—also proposed [3].

The North Jersey Community Research Initiative (NJCRI) has an HCV treatment program that has incorporated various parts of this framework into their methods to optimize treatment to marginalized communities, mainly persons who inject drugs (PWID) or who are unhoused.

In clinical practice, 2 commonly used medications that are pangenotypic are glecaprevir/pibrentasvir (GLE/PIB) and sofosbuvir/velpatasvir (SOF/VEL). Even though they are both safe and effective for the treatment of HCV infection, they have never been studied head-to-head in a randomized controlled trial to compare their efficacy and safety. Real-world data suggest that that they are similarly effective and safe in the general population, but they have not been compared within our unique model of delivery of care, which caters mainly to individuals in drug rehabilitation programs.

## METHODS

Data were collected from 05/01/2021 to 02/28/2023 in an ongoing prospective observational study. Patients were included if they tested positive for HCV infection and were prescribed GLE/PIB or SOF/VEL. Patients were excluded if they were pregnant, were <18 years old, had received any other prescription treatment for HCV, or had decompensated liver cirrhosis. We compared baseline characteristics and outcomes between the GLE/PIB and SOF/VEL treatment groups. As a measure of efficacy, sustained virologic resistance (SVR) at 12 weeks after completion of therapy was used, as was rates of loss to follow-up (LTFU).

The mobile HCV elimination clinic, sponsored by NJCRI, travels to individual substance use disorder treatment facilities in New Jersey on a regular schedule and provides services in designated areas both inside and outside the facilities. Patients are met in their own environments (eg, clinics, encampments, homes, specifically requested locations) and on their own time, allowing for a more trusted and personal engagement with our health care system. Patients voluntarily came to the mobile van to get a rapid HCV test, and if positive, baseline lab tests were obtained in accordance with American Association for the Study of Liver Diseases–Infectious Diseases Society of America HCV treatment guidelines [5]. During the initial interaction, education on HCV transmission, consequences, and treatment was provided while patients were registered and scheduled for a telehealth appointment with an infectious disease specialist. Appointments usually took place within 7 days and were rescheduled should the patient be unavailable. At the designated telehealth appointment, assessment for therapy was made and prescriptions were sent to a contracted specialty pharmacy. The pharmacy communicated directly with the clients to deliver their medications, usually within 1 week of the telehealth visit. Nurses tracked treatment completion and performed blood work as ordered in the telehealth visit. In addition, each patient received full case management services including insurance enrollment and assistance.

For patients who were found to have bridging/advanced fibrosis (F3) predicted by FibroSURE, or for whom platelets were <150 000 µL, a portable transient elastography was used to confirm fibrosis score. The treatment choice depended mainly on drug–drug interaction, patient preference, and third-party payor decision. Adverse events were monitored using a patient reporting model where the patients were asked to call if they thought they could be having any adverse event while on therapy, in addition to the clinical oversight at the distributing pharmacy. The study was approved by the NJCRI Institutional Review Board.

The chi-square statistic was calculated to compare the observed and expected frequencies, and the *P* values are reported in Table 1. We used measures of central tendency and variability to describe and summarize the characteristics of the study sample. The analyses were conducted using SAS software, version 9.4 [6].

**Table 1.** Characteristics and Outcomes of Participants

	GLE/PIB	SOF/VEL	*P* Value
Total, No. (%)	340 (44)	429 (56)	
Demographics			
Median age, y	40	43	.003
Male 481 (63%), No. (%)	222 (65)	259 (60)	.161
Female 287 (37%), No. (%)	117 (34)	170 (40)	.137
Genotype, No. (%)			
GT1a	213 (63)	269 (63)	.987
GT3	72 (21)	72 (17)	.120
Other GT	40 (12)	71 (17)	.060
Fibrosis, No. (%)			
F0-2	320 (94)	358 (83)	<.001
F3-4	19 (6)	69 (17)	<.001
Treatment, No.			Total
Completed treatment	313	382	695
Lost to follow-up	27	47	74
Total	340	429	769

Abbreviations: GLE/PIB, glecaprevir/pibrentasvir; SOF/VEL, sofosbuvir/velpatasvir.

## RESULTS

We enrolled 769 persons during the period of 05/01/2021 to 02/28/2023. Table 1 presents the distribution of study population characteristics. The study groups shared commonalities in age and gender (as recorded in the health record), with the median age being slightly higher in the SOF/VEL group, 43 years, compared with the GLE/PIB group, 40 years. There was no significant difference in the gender of the participants. Most individuals in both groups had genotype 1a. The only notable difference was in fibrosis scores, with the SOF/VEL group showing a higher grade (F3-4) compared with the GLE/PIB group, 17% to 5.6%, respectively (*P* < .001). The GLE/PIB group had more patients with F0-F2 fibrosis (*P* < .001).

Overall, of the 769 patients treated with either of these agents, 90.4% completed treatment and 9.6% were LTFU. Among the patients treated with GLE/PIB (n = 340), 92% completed treatment and 8% (n = 27) were LTFU. For patients treated with SOF/VEL (n = 429), 89% completed treatment and 11% (n = 47) were LTFU.

**Figure 1.**
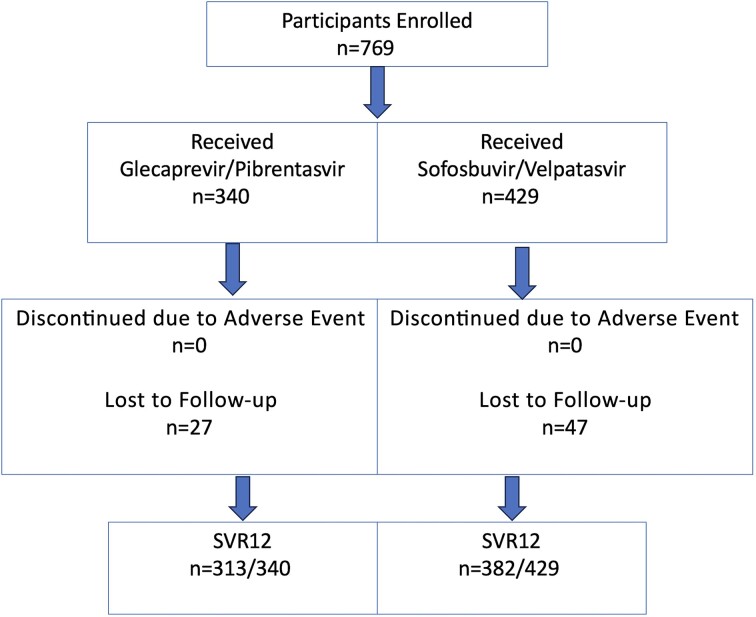
The outcome of study participants, describing those lost to follow-up and treatment completion. SVR12 is defined as sustained virologic response 12 weeks after therapy completion.

When comparing our calculated chi-square statistic with the critical value from the chi-square distribution table at 1 degree of freedom and a significance level of .05 (or 5%), we found no statistically significant differences in treatment completion rates between therapies. The most common drug–drug interactions were aripiprazole, quetiapine, and proton pump inhibitors (PPIs). Aripiprazole and quetiapine favored SOF/VEL, whereas PPI favored GLE/PIB. None of the participants who finished therapy had a virologic failure, and there was no documented re-infection (Figure 1).

## DISCUSSION

When treating HCV infection in persons with a history of SUD utilizing the unique treatment model of a medical mobile van with telehealth medicine, this study demonstrated no difference between GLE/PIB and SOF/VEL. However, treatment logistics might favor GLE/PIB compared with SOF/VEL. For example, therapy with GLE/PIB is recommended for a minimum of only 8 weeks as opposed to 12 weeks with SOF/VEL [7]. In the GLE/PIB group, only 8% were LTFU compared with 11% in the SOF/VEL group. The longer duration of therapy for SOF/VEL could be a potential explanation for the higher rate of LTFU compared with GLE/PIB. Both regimens are exceptionally well tolerated. In this study, no patients discontinued therapy due to adverse events, although it is unknown if side effects contributed to why someone might have been LTFU. This study demonstrates that if you are initiating therapy in this population, the ultimate clinically relevant end point of achieving SVR is not affected by the choice of DAA.

However, the generalizability of this study is somewhat limited by its design as a single-center, observational cohort. This study protocol may also have underestimated the true incidence of adverse events, as it relied solely on passive patient reporting without active surveillance.

The low rates of LTFU also suggest that a mobile HCV elimination program may be a useful tool in providing treatment to this population. This approach reflects many of the building blocks suggested by the WHO. Service delivery is one of the priorities of the mobile clinic, providing individuals with information and testing for HCV at SUD treatment facilities, avoiding additional appointments, and delivering care in a more convenient way. Service delivery is further promoted through follow-up visits with telemedicine and delivering medications by mail. Communication and engagement are encouraged through educating individuals about HCV while they are at the initial evaluation before testing. We found that this encourages individuals to receive testing for HCV and further empowers them to continue care after diagnosis. This project enhances health information systems by comparing the efficacy of 2 DAAs (GLE/PIB and SOF/VEL) in the treatment of HCV in this particular population. This clinic incorporates medical procurement by using rapid HCV testing and focuses on treatment regimens that are pangenotypic. This clinic also provides further follow-up testing including noninvasive liver disease screening at the time of diagnosis, which has been shown to increase rates of adherence [3].

By providing patient-centered care through a multidisciplinary approach with a focus on meeting individuals where they are, the mobile HCV elimination clinic is a way to improve diagnosis and treatment of HCV infection in persons with a history of SUD.

## CONCLUSIONS

With high rates of SVR and low rates of patient LTFU, the mobile HCV elimination clinic can be an effective strategy to reach people who inject drugs, those with unstable housing, and people with SUD. This mobile clinic focuses on meeting patients where they are, rapid testing for HCV, and organizing patient care with physicians, pharmacists, and case management. Our large real-world study showed no significant difference between GLE/PIB and SOF/VEL in efficacy or safety when treating HCV infection utilizing a unique treatment model of a mobile medical unit combined with telehealth, even when co-located with SUD treatment facilities.
